# Menopausal hormone therapy and risk of liver cancer in a Swedish population-based cohort study

**DOI:** 10.1093/jnci/djag084

**Published:** 2026-03-22

**Authors:** Yongying Huang, Giola Santoni, Jessica L Petrick, Victoria Wocalewski, Ernesto Sparrelid, Shao-Hua Xie

**Affiliations:** Clinical Oncology School of Fujian Medical University, Fujian Cancer Hospital, Fuzhou, China; Upper Gastrointestinal Surgery, Department of Molecular Medicine and Surgery, Karolinska Institutet, Stockholm, Sweden; Upper Gastrointestinal Surgery, Department of Molecular Medicine and Surgery, Karolinska Institutet, Stockholm, Sweden; Slone Epidemiology Center at Boston University, Boston, MA, United States; Upper Gastrointestinal Surgery, Department of Molecular Medicine and Surgery, Karolinska Institutet, Stockholm, Sweden; Division of Surgery and Oncology, Department of Clinical Science, Intervention and Technology, Karolinska Institutet, Karolinska University Hospital, Stockholm, Sweden; Upper Gastrointestinal Surgery, Department of Molecular Medicine and Surgery, Karolinska Institutet, Stockholm, Sweden; School of Public Health, Fujian Medical University, Fuzhou, China

## Abstract

**Background:**

Previous studies have suggested a potential role of sex hormones in the development of liver cancer. This study aimed to examine whether menopausal hormone therapy (MHT) is associated with a decreased risk of liver cancer by histological type.

**Methods:**

This Swedish population-based cohort study included 217 878 women who received MHT in 2006 to 2023 and an age-matched comparison group of 1 089 390 women who did not receive MHT. Cox regression assessed the associations between use of MHT and the risk of 2 main subtypes of liver cancer, that is, hepatocellular carcinoma and intrahepatic cholangiocarcinoma, with adjustment for smoking- and alcohol-related diagnoses, non-alcoholic fatty liver disease, diabetes or obesity, hysterectomy, use of non-steroidal anti-inflammatory drugs or aspirin, and use of statins.

**Results:**

MHT users had a decreased risk of hepatocellular carcinoma (hazard ratio [HR] = 0.46, 95% confidence interval [CI] = 0.30 to 0.72). Decreased HRs of hepatocellular carcinoma were indicated both in users of estrogen only (HR = 0.42, 95% CI = 0.21 to 0.86) and estrogen combined with progestogen (HR = 0.49, 95% CI = 0.28 to 0.85). The risk reduction in hepatocellular carcinoma was apparently more pronounced in users aged 60 years or older (HR = 0.37, 95% CI = 0.19 to 0.75). The use of MHT was not associated with the risk of intrahepatic cholangiocarcinoma (HR = 0.99, 95% CI = 0.72 to 1.36).

**Conclusions:**

MHT in women may decrease the risk of hepatocellular carcinoma, but not intrahepatic cholangiocarcinoma.

## Background

Liver cancer is the sixth most commonly diagnosed cancer and the third leading cause of cancer-related deaths worldwide in 2022, with hepatocellular carcinoma (HCC, accounting for 75%–85% of all cases) and intrahepatic cholangiocarcinoma (iCCA, 10%–15% of all cases) being the predominate types.^1^ HCC and iCCA share several major risk factors, including chronic hepatitis B or C virus infection, heavy alcohol consumption, type 2 diabetes, and obesity. Subtype-specific risk factors include dietary aflatoxin exposure for HCC, and liver flukes, primary sclerosing cholangitis, and inflammatory bowel diseases for iCCA.^1-4^

Liver cancer is more common in men than in women, with the male-to-female incidence ratio ranging from 2 to 4 for HCC, but only 1 to 2 for iCCA in most countries.^5-7^ Such sex difference may be partially attributed to a higher prevalence of hepatitis virus infection, tobacco smoking, and alcohol overconsumption in men. However, an even more striking male predominance has been observed in mouse models of HCC induced by the carcinogenic agent diethylnitrosamine, implying that some intrinsic differences between the sexes may play a role in the development of HCC.^2^^,^^8^ Specifically, sex hormonal exposures have been hypothesized to be involved in the development of liver cancer, particularly a protective role of female sex hormones.^9^ However, the existing evidence from epidemiological studies remains limited and inconclusive.^9-14^

Menopausal hormone therapy (MHT), also known as hormone replacement therapy, is a medication containing estrogen only or estrogen combined with progestogen in the treatment of postmenopausal symptoms in women. A few earlier studies have investigated the association between use of MHT and the risk of liver cancer, but the findings have been conflicting and most of them did not separate histological types or assessed HCC only.^13^^,^^15-19^

This population-based study aimed to examine whether use of MHT is associated with a decreased risk of liver cancer by histological type in a population with a low prevalence of hepatitis B or C virus infection.

## Patients and methods

### Study design and participants

This was a nationwide population-based cohort study. The source population consisted of all female Swedish residents aged 45 years or older who were included in the Swedish Prescribed Drugs and Health Cohort (SPREDH), details of which have been described previously.^20^^,^^21^ The most recently up-dated version of SPREDH includes more than 9 million Swedish residents (the vast majority of the Swedish population) with dispensed records of selected commonly prescribed medications during the period between July 1, 2005 and December 31, 2023, as recorded in the Swedish Prescribed Drugs Registry. The SPREDH also contains relevant health data of the participants, retrieved from 3 other national registries in Sweden, that is, the Cancer Registry, the Patient Registry, and the Cause of Death Registry. All these registries have complete nationwide coverage,^22-25^ and the unique personal number enables the linkage between these registries.

Individuals were excluded if they had: (1) at least 2 records of MHT dispensation before the age of 45 years or before July 1, 2006 in the Prescribed Drug Registry; (2) diagnosis of liver cancer, breast cancer, or gynecologic cancer before cohort entry as recorded in the Cancer Registry; (3) prior diagnosis of viral hepatitis in the Patient Registry; or (4) prior history of oophorectomy in the Patient Registry. The corresponding Anatomical Therapeutic Chemical (ATC) classification codes for MHT, and codes for these diagnoses and surgical procedures, are illustrated in Tables S1–S4. All participants were followed up until any of the study outcomes, death, or the end of the study (December 31, 2023), whichever occurred first.

This study was approved by the Regional Ethical Review Board in Stockholm, Sweden (reference number 2016/982-31/4). The study was conducted conforming to the Declaration of Helsinki. Informed consent is not required for registry-based research in Sweden.

### Exposure

The exposure was at least 2 dispensed records of systemic MHT (oral, transdermal, or injection), either estrogen only or estrogen combined with progestogen, at an interval of over 1 month and < 1.5 years, during the study period between July 1, 2006 and December 31, 2023. Dispensation of local MHT (vaginal form) was not included. We excluded those who started use of MHT during the first year (July 2005 to June 2006) after the implantation of the Swedish Prescribed Drug Registry, that is, “prevalent” users, and thus, we assumed that this study included “incident” (new) users of MHT only because a prescription is valid for up to 12 months in Sweden. The MHT medications are identified from the Swedish Prescribed Drug Registry by their ATC codes (Table S1). The exposure started at the time of the second purchase of MHT. We did not define the exposure as starting at the first MHT prescription because we intended to avoid including short-term users who stopped the treatment because of side effects or other reasons, where the influence of cancer risk would be minimal or absent. For each MHT user, 5 age-matched (±1 year) female non-users were randomly selected from SPREDH on the date of the user’s second MHT dispensation, serving as the comparison (unexposed) group. If <20 women were available for the matching, the age range would be increased by 1 year until at least 20 women were available for matching. The exposure was treated as a time-varying variable; participants remained “unexposed” if they had only one dispensation and were shifted to the exposed group at any second dispensation of MHT.

### Outcome

The study outcome was newly diagnosed liver cancer as recorded in the Cancer Register. We separately assessed the 2 major subtypes of liver cancer, that is, HCC (diagnosis code C22.0 in the International Classification of Diseases, 10th edition [ICD-10]) and iCCA (ICD-10 code C22.1). Censoring for death was ascertained through linkage to the Cause of Death Register.

### Covariates

Several covariates were considered in the analyses, including age at cohort entry (continuous), calendar year at cohort entry (continuous), smoking-related diagnoses (yes or no), alcohol-related diagnoses (yes or no), non-alcoholic fatty liver disease (NAFLD) (yes or no), obesity or diabetes (yes or no), hysterectomy (yes or no), use of non-steroidal anti-inflammatory drugs (NSAIDs) or aspirin (yes or no), and use of statins (yes or no). Because obesity is likely under-reported in the Patient Registry, we used a combined covariate of obesity and diabetes as a proxy. Diagnoses and history of hysterectomy before cohort entry were searched for in the Patient Register. The use of other medications (NSAIDs or aspirin, and statins) was defined as at least 2 dispensed records within 1 year before cohort entry in the Prescribed Drug Registry. The codes used to identify the covariates are provided in Tables S3–S5.

### Statistical analysis

Cox proportional hazards regression, with follow-up time as the time metric, was performed to assess the relative risk of liver cancer in MHT users compared to non-users, providing hazard ratios (HR) with 95% confidence intervals (CI). The Cox model allowed baseline hazards to differ by year of birth, but the coefficients were constrained to be the same across strata. Two models were applied, that is, a crude model with adjustment for age and calendar year and a multivariable model with additional adjustment for all the covariates mentioned above.^26^ We assessed the dose–response relation among users of MHT by categorizing the dosage into 3 approximately equal-sized groups according to the total daily defined dose (DDD) in the first year after cohort entry, that is, <210 DDDs, 210 to 420 DDDs, and >420 DDDs. *P* value for trend was tested by treating the dosage as a continuous variable.

We conducted subgroup analyses by (1) the regimen of MHT (estrogen only, or estrogen combined with progestin); (2) age at cohort entry (≤60, or >60 years); and (3) duration of follow-up (≤5 years or >5 years). The cut-off of 60 years in subgroup analysis by age was selected a priori based on biological considerations related to menopausal status and age-related hormonal changes. Sensitivity analyses were conducted by (1) excluding those with any previous diagnosis of cancers except for non-melanoma skin cancer; (2) excluding cases occurring within the first year of follow-up; (3) excluding those with previous liver transplant; (4) eliminating covariates of diabetes or obesity, and NAFLD from the multivariable model separately, considering the possible involvement of these factors in the associations between sex hormone exposures and liver cancer; and (5) with additional adjustment for previous diagnosis of liver cirrhosis.

Date management and statistical analyses were performed using Stata (Release 15, StataCorp, College Station, TX, United States) by 1 author (Y.H.) following a detailed study protocol and validated by an experienced biostatistician (G.S.). All analyses are 2-sided and a *P* value < .05 was considered statistically significant.

## Results

### Participants

This study included 217 878 users of MHT (exposed) and 1 089 390 women in the age-matched comparison group (unexposed). The detailed selection of the participants is displayed in Figure S1. Characteristics of participants are summarized in detail in Table 1. The mean age at cohort entry was 55.5 (± 9.9 standard deviation) years. The prevalence of smoking-related diagnoses, alcohol-related diagnoses, NAFLD, use of NSAIDs or aspirin, and use of statins was similar in both groups. The prevalence of diabetes or obesity was slightly lower in users of MHT (5.7%) than in non-users (6.8%) of MHT, while the proportion of prior hysterectomy was slightly higher in users (5.7%) than in non-users (3.1%). Among users of MHT, approximately one third used estrogen only and the remaining two thirds used estrogen combined with progestogen (Table 1). The median duration (interquartile range) of follow-up was 6.0 (2.5-12.2) years in the exposed group and 5.5 (2.2-11.6) years in the comparison group.

**Table 1.** Characteristic of users and non-users of menopausal hormone therapy, No. (%).

	Users	Non-users
Total	217 878 (100)	1 089 390 (100)
Age, years		
45 to 49	44 776 (20.6)	223 880 (20.6)
50 to 54	95 889 (44.0)	479 445 (44.0)
55 to 59	42 656 (19.6)	213 280 (19.6)
≥60	34 557 (15.9)	172 785 (15.9)
Mean ± standard deviation	55.5 ± 9.9	55.5 ± 9.9
Smoking-related diagnoses	3550 (1.6)	18 994 (1.7)
Alcohol-related diagnoses	4468 (2.1)	22 006 (2.0)
Non-alcoholic fatty liver disease	180 (0.1)	1360 (0.1)
Diabetes or obesity	12 428 (5.7)	73 822 (6.8)
Liver cirrhosis	162 (0.1)	1410 (0.1)
Hysterectomy	12 429 (5.7)	33 627 (3.1)
Liver transplant	20 (<0.1)	165 (<0.1)
Use of non-steroidal anti-inflammatory drugs or aspirin	14 323 (6.6)	69 538 (6.4)
Use of stains	3340 (1.5)	23 067 (2.1)
Death during follow-up	19 682 (9.0)	97 582 (9.0)
Menopausal hormone therapy regimen		
Estrogen only	78 718 (36.1)	
Estrogen plus progestogen	139 160 (63.9)	

### Risk of hepatocellular carcinoma

**Figure 1.**
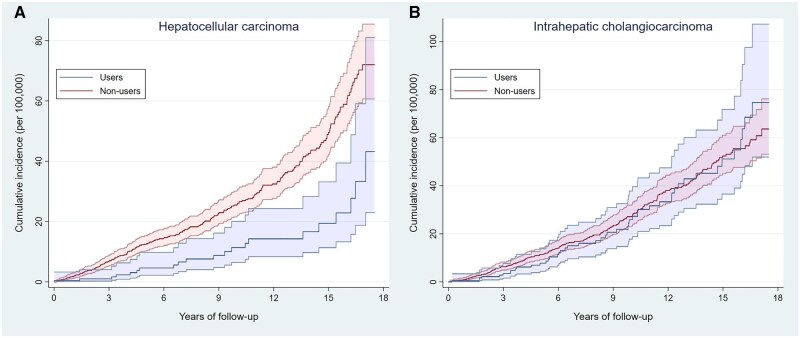
Cumulative incidence of **(A)** hepatocellular carcinoma and **(B)** intrahepatic cholangiocarcinoma among users and non-users of menopausal hormonal therapy during follow-up.

During the follow-up, 21 users of MHT (in 1 613 174 person-years) and 232 non-users (in 7 693 909 person-years) were diagnosed with HCC. MHT users had a decreased risk of HCC (adjusted HR = 0.46, 95% CI = 0.30 to 0.72) (Figure 1A and Table 2). Decreased HRs of HCC were indicated both in users of estrogen only (HR = 0.42, 95% CI = 0.21 to 0.86) and estrogen combined with progestogen (HR = 0.49, 95% CI = 0.28 to 0.85) (Table 2). No clear dose–response relation was found for the risk of HCC associated with MHT use (Table 3). The risk reduction in HCC was more pronounced in users aged 60 years or older (HR = 0.37, 95% CI = 0.19 to 0.75) and those who were followed up for ≤ 5 years (HR = 0.37, 95% CI = 0.17 to 0.80) (Table 3).

**Table 2.** Hazard ratios (HRs) with 95% confidence intervals (CIs) for associations between use of menopausal hormone therapy and the risk of liver cancer.

Outcome and exposure	Number of cases	Person-years	Crude HR (95% CI)^a^	Adjusted HR (95% CI)^b^
Hepatocellular carcinoma				
Non-users	232	7 693 909	1.00 (Reference)	1.00 (Reference)
Users	21	1 613 174	0.45 (0.29 to 0.70)	0.46 (0.30 to 0.72)
Users of estrogen only	8	511 208	0.41 (0.20 to 0.83)	0.42 (0.21 to 0.86)
Users of estrogen combined with progestogen	13	1 101 966	0.47 (0.27 to 0.83)	0.49 (0.28 to 0.85)
Intrahepatic cholangiocarcinoma				
Non-users	227	7 693 909	1.00 (Reference)	1.00 (Reference)
Users	47	1 613 174	1.00 (0.73 to 1.37)	0.99 (0.72 to 1.36)
Users of estrogen only	16	511 208	0.95 (0.57 to 1.59)	0.90 (0.53 to 1.53)
Users of estrogen combined with progestogen	31	1 101 966	1.03 (0.70 to 1.50)	1.04 (0.71 to 1.53)

a Adjusted for age and calendar year.

b Further adjusted for smoking-related diagnoses, alcohol-related diagnoses, non-alcoholic fatty liver disease, diabetes or obesity, hysterectomy, use of non-steroidal anti-inflammatory drugs or aspirin, and use of statins.

**Table 3.** Hazard ratios (HRs) with 95% confidence intervals (CIs) for associations between use of menopausal hormone therapy and the risk of liver cancer stratified by age, follow-up period, and dosage.

	Person-years	Hepatocellular carcinoma			Intrahepatic cholangiocarcinoma		
		Number of cases	Crude HR (95% CI)^a^	Adjusted HR (95% CI)^b^	Number of cases	Crude HR (95% CI)^a^	Adjusted HR (95% CI)^b^
Age							
≤60 years							
Non-users	6 304 475	106	1.0 (Reference)	1.0 (Reference)	141	1.0 (Reference)	1.0 (Reference)
Users	1 347 814	12	0.52 (0.29 to 0.95)	0.55 (0.30 to 0.99)	33	1.08 (0.74 to 1.58)	1.07 (0.74 to 1.57)
>60 years							
Non-users	1 389 434	126	1.0 (Reference)	1.0 (Reference)	86	1.0 (Reference)	1.0 (Reference)
Users	265 360	9	0.37 (0.19 to 0.73)	0.37 (0.19 to 0.75)	14	0.84 (0.48 to 1.49)	0.83 (0.47 to 1.47)
Follow-up							
≤5 years							
Non-users	4 039 120	98	1.0 (Reference)	1.0 (Reference)	89	1.0 (Reference)	1.0 (Reference)
Users	827 200	7	0.37 (0.17 to 0.79)	0.37 (0.17 to 0.80)	11	0.62 (0.33 to 1.15)	0.61 (0.33 to 1.14)
>5 years							
Non-users	3 654 789	134	1.0 (Reference)	1.0 (Reference)	138	1.0 (Reference)	1.0 (Reference)
Users	785 974	14	0.50 (0.29 to 0.87)	0.53 (0.30 to 0.91)	36	1.24 (0.86 to 1.79)	1.23 (0.85 to 1.78)
Dosage^c^							
≤210 DDD	576 011	8	1.0 (Reference)	1.0 (Reference)	20	1.0 (Reference)	1.0 (Reference)
210-420 DDD	509 070	7	1.16 (0.41 to 3.26)	1.22 (0.43 to 3.43)	11	0.69 (0.33 to 1.45)	0.70 (0.33 to 1.47)
≥420 DDD	515 820	5	1.02 (0.31 to 3.31)	1.12 (0.34 to 3.67)	15	1.09 (0.54 to 2.21)	1.12 (0.55 to 2.28)
*P* for trend			0.495	0.418		0.866	0.898

a Adjusted for age and calendar year.

b Further adjusted for smoking-related diagnoses, alcohol-related diagnoses, non-alcoholic fatty liver disease, diabetes or obesity, hysterectomy, use of non-steroidal anti-inflammatory drugs or aspirin, and use of statins.

c Total daily defined dose (DDD) in the first year after cohort entry.

### The risk of intrahepatic cholangiocarcinoma

During the follow-up, 47 users of MHT and 227 non-users developed iCCA. The use of MHT was not associated with the risk of iCCA (adjusted HR=  0.99, 95% CI = 0.72 to 1.36) (Figure 1B, Table 2). The HRs of iCCA were 0.90 (95% CI = 0.53 to 1.53) for estrogen only and 1.04 (95% CI = 0.71 to 1.53) for estrogen combined with progestogen (Table 2). The use of MHT was not associated with the risk of iCCA in the subgroup analyses, except for a seemingly inverse association, without statistical significance, in participants who were followed up for ≤5 years (HR = 0.61, 95% CI = 0.33 to 1.14) (Table 3).

### Sensitivity analyses

The sensitivity analyses excluding those with previous cancer diagnosis, cases occurring within the first year of follow-up, or those with previous liver transplant, eliminating the covariates of diabetes or obesity and NAFLD from the multivariable model, or with additional adjustment for liver cirrhosis, did not substantially change the risk estimates (Table 4).

**Table 4.** Hazard ratios (HRs) with 95% confidence intervals (CIs) for associations between use of menopausal hormone therapy and the risk of liver cancer in sensitivity analyses.

Sensitivity analyses	Person-years	Hepatocellular carcinoma		Intrahepatic cholangiocarcinoma	
		Number of cases	Adjusted HR (95% CI)^a^	Number of cases	Adjusted HR (95% CI)^a^
Excluding individuals with previous cancer diagnosis					
Non-users	7 470 225	224	1.0 (Reference)	219	1.0 (Reference)
Users	1 567 817	20	0.45 (0.29 to 0.72)	47	1.03 (0.75 to 1.41)
Excluding cases occurring within 1 year of follow-up					
Non-users	7 693 890	214	1.0 (Reference)	215	1.0 (Reference)
Users	1 613 172	20	0.46 (0.30 to 0.73)	46	1.01 (0.73 to 1.38)
Excluding individuals with previous liver transplant					
Non-users	7 692 990	231	1.0 (Reference)	227	1.0 (Reference)
Users	1 613 031	20	0.43 (0.27 to 0.67)	47	0.97 (0.71 to 1.33)
Not adjusting for non-alcoholic fatty liver disease					
Non-users	7 693 909	232	1.0 (Reference)	227	1.0 (Reference)
Users	1 613 174	21	0.46 (0.30 to 0.72)	47	0.99 (0.72 to 1.36)
Not adjusting for obesity or diabetes					
Non-users	7 693 909	232	1.0 (Reference)	227	1.0 (Reference)
Users	1 613 174	21	0.45 (0.29 to 0.71)	47	0.99 (0.72 to 1.36)
Additional adjusting for liver cirrhosis					
Non-users	7 693 909	232	1.0 (Reference)	227	1.0 (Reference)
Users	1 613 174	21	0.48 (0.31 to 0.75)	47	1.00 (0.73 to 1.37)

a Adjusted for age, calendar year, smoking-related diagnoses, alcohol-related diagnoses, non-alcoholic fatty liver disease, diabetes or obesity, hysterectomy, use of non-steroidal anti-inflammatory drugs or aspirin, and use of statins.

## Discussion

This population-based cohort study suggests a decreased risk of HCC in women using MHT, which is consistent for either estrogen only or combined with progestogen. No association was found between use of MHT and the risk of iCCA. Such findings were robust across various subgroup analyses and sensitivity analyses.

Strengths of this study included the population-based cohort design, the use of high-quality data from registries with complete nationwide coverage, large sample size, documented prescription data, and the long and complete follow-up. We used a new-user vs non-user design, which ensured clear temporality between covariates and exposure initiation whilst avoiding bias due to the inclusion of prevalent users.^27^ We also excluded those with only 1 dispensation record within the defined period, that is, short-term users of MHT, which would have reduced the chance of exposure misclassification and confounding by indication or contraindication. The various subgroup and sensitivity analyses substantiated the validity of the findings and explored heterogeneity between groups.

This study also has some limitations. First, due to the lack of data on lifestyle risk factors, such as tobacco smoking and alcohol use, we used related diagnoses as proxies for these potential confounders. In addition, some of the covariates, particularly obesity, were likely underreported in the registries. Therefore, residual confounding cannot be ruled out, although we attempted to account for it by using a combined covariate of obesity and obesity as proxy. Second, because of the low prevalence of hepatitis viral infection (<2% to 3%) in the Swedish population, we excluded individuals with viral hepatitis. This should have, to some extent, reduced potential confounding by hepatitis viral infection, but undiagnosed infections may remain. A pooled analysis of 11 cohort studies (the Liver Cancer Pooling Project) in the United States assessed serologic markers for hepatitis viral infection in a subset of participants and the results suggested minimal influence of residual confounding from viral hepatitis on the observed association.^18^ However, information on such serologic markers or antiviral treatment was not available in the present study. In addition, exclusion of individuals with a prior diagnosis of viral hepatitis might have limited the generalizability of our findings to populations in which hepatitis viral infection is a substantial etiological factor. Third, there were no data on reproductive factors that may influence sex hormone levels. Lastly, due to the low incidence of liver cancer in women, the statistical power remained limited, at least for iCCA, for which the strength of association, if any, may be weak. However, to the best of our knowledge, this was the largest study to date examining the association between MHT and iCCA. Although the inverse association between MHT use and HCC was statistically significant and consistent across MHT formulations and age groups, further studies with larger numbers of exposed cases are warranted for more precise estimates of the association.

A few studies have previously assessed the association between MHT use and the risk of liver cancer, but many of these studies did not separate histological types,^15-17^ which have several different etiological factors and notably dissimilar sex difference. The observed decreased risk of HCC in MHT users in the present study was in line with most of the previous studies specifically investigating HCC. An earlier case-control study in Taiwan found a decreased risk of HCC associated with MHT use, possibly restricted to hepatitis B surface antigen-negative individuals.^19^ Cohort studies in Taiwan of women, all infected with hepatitis virus B or C, also reported approximately 50% decreased risk of HCC in MHT users compared with propensity score matched non-users.^28^^,^^29^ A hospital-based case-control study in the United States found a reduced risk of HCC, including hepatitis-related tumor, associated with use of estrogen MHT.^30^ In contrast, a pooled analysis in the Liver Cancer Pooling Project found a modestly increased risk of HCC associated with MHT use (HR = 1.35, 95% CI = 1.01 to 1.81).^18^ Such inconsistency may be explained by differences across studies in study design, particularly controlling for confounding by oophorectomy and hepatitis viral infection, population heterogeneity, and chance. To the best of our knowledge, only 1 study has previously investigated the association between MHT use and the risk of iCCA.^13^ This pooled-analysis of 12 North American-based cohort studies (in the Liver Cancer Pooling Project) and the UK Biobank found no decreased risk of iCCA in MHT users (HR = 1.10, 95% CI = 0.82 to 1.49),^13^ which was consistent with the current study.

The apparently stronger association for HCC observed among older new users and participants with shorter follow-up, 2 groups that largely overlapped, may reflect greater biological relevance of exogenous hormone exposure in the postmenopausal period when endogenous estrogen levels are markedly reduced. However, as inverse associations were also observed across other age and follow-up strata, these subgroup differences should be interpreted cautiously and not as evidence of effect modification.

The potential protective effect of estrogen against HCC may be due to the anti-inflammatory properties of estrogen. Estrogen can bind to estrogen receptor α (ERα) located in the nucleus of Kupffer cells in the liver, and then inhibit the production of interleukins to exert its anticarcinogenic effect by decreasing the activity of transcription factors, including nuclear factor κB (NF-κB), signal transducer and activator of transcription 3 (STAT3), and CCAAT/enhancer-binding protein β (C/EBPβ).^12^^,^^31^ The protective effect of estrogen may also be attributed to the antiproliferative and anti-inflammatory properties through binding to and activation of estrogen receptor beta (ERβ).^32^^,^^33^ In the current study, no difference was observed in risk estimates with and without adjustment for NAFLD and diabetes or obesity, implying that the effect of MHT may be independent of these conditions.

The findings of this study, together with previous lines of evidence, highlight the need for further investigating whether MHT has a risk reduction effect against HCC. However, considering the low incidence of HCC in the general population, randomized controlled trials may be possible in selected high-risk populations only and still require large collaborative endeavors. Despite the probably protective potential of MHT against HCC as well as other gastrointestinal cancers,^34^^,^^35^ the existing evidence is far from enough to influence the current clinical practice, which needs to balance the benefits and risk of harms for individual patients, including increased risk of other cancer types, for example, breast and ovarian cancer.^34^

In summary, this large nationwide population-based cohort study suggested that MHT use in women is associated with a decreased risk of HCC but not iCCA, in a western population with a low prevalence of hepatitis viral infection. These findings support the hypothesis that sex hormones contribute to the gender disparity in the incidence of HCC and not of iCCA.

## Supplementary Material

djag084_Supplementary_Data

## Data Availability

All the data in this study were retrieved from the Swedish Prescribed Drugs and Health Cohort. The original data are available from the related registries listed above but restrictions apply to the availability of these data, which were used under license for this study and therefore are not publicly available. The data are, however, available through application to these registries or reasonable request to the corresponding author (S.H.X.).
