# Supplementary material for: Menopausal hormone therapy and risk of liver cancer in a Swedish population-based cohort study
Source: J Natl Cancer Inst. 2026 Mar 22;118(8):1478–84. doi: 10.1093/jnci/djag084 (PMC13453530; doi:10.1093/jnci/djag084)
Supplement: Supplementary file 1 [file djag084_supplementary_data.docx]

**Menopausal hormone therapy and risk of liver cancer in a Swedish population-based cohort study**

**Supplementary Materials**

**Table S1.** Anatomical Therapeutic Chemical (ATC) classification codes for menopausal hormone therapy

**Table S2.** International Classification of Diseases (ICD) codes in Cancer Register

**Table S3.** Swedish version of the International Classification of Diseases (ICD) codes for medical histories in the Swedish Patient Register

**Table S4.** Nordic Medico-Statistical Committee codes for hysterectomy and oophorectomy in the Swedish Patient Registry

**Table S5.** ATC codes for prescribed drugs in the Swedish Prescribed Drug Registry

**Figure S1.** Flowchart of selection of users and non-users of menopausal hormone therapy (MHT) in the Swedish Prescribed Drug and Health Cohort

**Authors:** Yongying Huang, MD, MPH^1, 2^, Giola Santoni, PhD^2^, Jessica L. Petrick, MPH, PhD ^3^, Victoria Wocalewski, MD^2^, Ernesto Sparrelid, MD, PhD ^4^, Shao-Hua Xie, MD, PhD ^2, 5^

**Affiliations:**

^1^ Clinical Oncology School of Fujian Medical University, Fujian Cancer Hospital, Fuzhou, China

^2^ Upper Gastrointestinal Surgery, Department of Molecular Medicine and Surgery, Karolinska Institutet, Stockholm, Sweden

^3^ Slone Epidemiology Center at Boston University, Boston, MA, United States

^4^ Division of Surgery and Oncology, Department of Clinical Science, Intervention and Technology, Karolinska Institutet, Karolinska University Hospital, Stockholm, Sweden

^5^ School of Public Health, Fujian Medical University, Fuzhou, China.

**Corresponding author:** Shao-Hua Xie, MD, PhD, Upper Gastrointestinal Surgery, Department of Molecular medicine and Surgery, Karolinska Institutet, Blombäcks väg 23, 4^th^ floor, 17177 Stockholm, Sweden. Email: [shaohua.xie@ki.se](mailto:shaohua.xie@ki.se), Tel: +46 852484184

**Table S1.** Anatomical Therapeutic Chemical (ATC) classification codes for menopausal hormone therapy ^a^

| **Type** | **ATC codes** |
| --- | --- |
| Oestrogen | G03CA, G03CB, G03CC |
| Oestrogen and progestogen | G03CX, G03F |
| Progestogen | G03D |

^a^ G03CX (includes tibolone), G03F, or any kind of oestrogen with G03D were regarded as oestrogen combined with progestogen. Local menopausal hormone therapy (vaginal form) was excluded.

**Table S2.** International Classification of Diseases (ICD) codes in Cancer Register

| **Cancer type** | **ICD-7** | **ICD-10** |
| --- | --- | --- |
| Liver cancer | 155.0, 156 | C22 |
| Hepatocellular carcinoma | 155.0 | C22.0 |
| Intrahepatic cholangiocarcinoma | 155.0 | C22.1 |
| Breast cancer | 170 | C50 |
| Malignant neoplasms of female genital organs | 171-176 | C51-C58 |
| Cancers except for non-melanoma skin cancer | 140-209, excluding 191 | C00-C97, excluding C44 |

**Table S3.** Swedish version of the International Classification of Diseases (ICD) codes for medical histories in the Swedish Patient Register

| **Diagnoses** | **ICD-9 (1987-1996)** | **ICD-10 (1997 and onwards)** |
| --- | --- | --- |
| Viral hepatitis | 070 | B15-B19  Z22.5 (Hepatitis virus carrier) |
| Diabetes | 250 | E10-E14 |
| Obesity | 278A | E66 |
| Non-alcoholic fatty liver disease | 571W | K76.0 |
| Smoking-related diseases | 305B Tobacco overconsumption  490, 491, 492, 494, 496 Chronic obstructive pulmonary disease related diagnosis | Z72.0 Tobacco use;  J40-J44, J47 Chronic obstructive pulmonary disease related diagnosis;  F17 Psychiatric and addiction related diagnoses caused by tobacco consumption. |
| Alcohol-related conditions | 291, 303, V79B Alcohol-related disorders;  305A Alcohol abuse;  357F Alcohol polyneuropathy; 359E Alcoholic myopathy;  425F Alcohol cardiomyopathy; 535D Alcoholic gastritis;  571, 572W Alcoholic liver disease;  577B Alcohol-induced chronic pancreatitis;  790D Increased alcohol serum levels;  977D overdose using drugs used to support treatment of chronic alcoholism  980A, 980B, 980X Alcohol poisoning  V65E Alcohol abuse counselling and surveillance | F10 Alcohol-related disorders;  G31.2 Degeneration of nervous system due to alcohol;  G62.1 Alcohol polyneuropathy;  G72.1 Alcoholic myopathy;  I42.6 Alcohol cardiomyopathy; K29.2 Alcoholic gastritis;  K70 Alcoholic liver disease;  K86.0 Alcohol-induced chronic pancreatitis;  Z71.4 Alcohol abuse counselling and surveillance;  T51.0, T51.1, T51.9 Alcohol poisoning  Z50.2 Rehabilitation of alcohol abusers  Z71.4 Counseling and monitoring for alcohol abuse  Z72.1 Alcohol use |
| Liver cirrhosis | 571C Alcohol-related cirrhosis  571G Biliary cirrhosis  571F Other and unspecified liver cirrhosis | K70.3 Alcohol-related cirrhosis  K74.3, K74.4 Biliary cirrhosis  K74.6 Other and unspecified liver cirrhosis |

**Table S4.** Nordic Medico-Statistical Committee codes for hysterectomy and oophorectomy in the Swedish Patient Registry

| Procedure | Before 1997 | 1997 and onwards |
| --- | --- | --- |
| Hysterectomy | 7220-7223, 7228 | LCD |
| Oophorectomy | 7020-7022, 7030-7032 | LAE, LAF |
| Liver transplant | 5200, 5202 | JJC |

**Table S5.** ATC codes for prescribed drugs in the Swedish Prescribed Drug Registry

| Medication | ATC codes |
| --- | --- |
| NSAIDS or aspirin | M01A, M02AA, N02BA, N02AJ, S01BC, S01CC, A01AD05, B01AC06, B01AC56, B01AF51, C07FX02, C07FX03, C07FX04, C10BX01, C10BX02, C10BX04, C10BX05, C10BX06, C10BX08, C10BX12, C01EB16, G02CC01, M01BA03, R02AX02 |
| Statins | C10AA, C10B |

**Figure S1.** Flowchart of selection of users and non-users of menopausal hormone therapy (MHT) in the Swedish Prescribed Drug and Health Cohort
